# MEG studies of motor cortex gamma oscillations: evidence for a gamma “fingerprint” in the brain?

**DOI:** 10.3389/fnhum.2013.00575

**Published:** 2013-09-17

**Authors:** Douglas Cheyne, Paul Ferrari

**Affiliations:** ^1^Program in Neurosciences and Mental Health, Hospital for Sick Children Research InstituteToronto, ON, Canada; ^2^Radiology Department, Meadowlands Hospital Medical CenterSecaucus, NJ, USA

**Keywords:** MEG, gamma oscillations, motor cortex, frequency tuning, basal ganglia

## Abstract

The human motor cortex exhibits transient bursts of high frequency gamma oscillations in the 60–90 Hz range during movement. It has been proposed that gamma oscillations generally reflect local intracortical activity. However, movement-evoked gamma is observed simultaneously in both cortical and subcortical (basal ganglia) structures and thus appears to reflect long-range cortical-subcortical interactions. Recent evidence suggests that gamma oscillations do not simply reflect sensory reafference, but have a facilitative role in movement initiation. Here we summarize contributions of MEG to our understanding of movement-evoked gamma oscillations, including evidence that transient gamma bursts during the performance of specific movements constitutes a stereotyped spectral and temporal pattern within individuals—a gamma “fingerprint”—that is highly stable over time. Although their functional significance remains to be fully understood, movement-evoked gamma oscillations may represent frequency specific tuning within cortical-subcortical networks that can be monitored non-invasively using MEG during a variety of motor tasks, and may provide important information regarding cortical dynamics of ongoing motor control.

Cortical oscillations in the frequency range of 30–90 Hz—so called *gamma band* oscillations—can be recorded from a wide range of brain regions during rest or the performance of cognitive-motor tasks, and are assumed to reflect wide-scale neuronal processes associated with cognition and perception (Tallon-Baudry and Bertrand, 1999; Fries, 2009). Such oscillations are variably time-locked to specific sensory or motor events and such “induced” gamma oscillations have been proposed to reflect global brain mechanisms underlying the attentional control of sensory input (Womelsdorf and Fries, 2007). Oscillations in the lower gamma range can also be elicited or “evoked” by sensory stimulation with modality specific effects, and have been implicated in the binding of low level input within sensory systems that underlies feature processing, particularly in the visual system (Engel and Singer, 2001). Magnetoencephalography (MEG) studies have shown that gamma band oscillations can also be recorded macroscopically from various sensory and motor areas. For example, gamma activity in the 40 Hz range can be observed in auditory areas during transient or steady-state acoustic stimulation (Pantev et al., 1991; Ross et al., 2005) and speech (Palva et al., 2002). MEG studies have shown sustained gamma oscillations in the 30–60 Hz range in primary visual cortex during presentation of contrast gratings, which are modulated by a variety of stimulus parameters, such as eccentricity, spatial frequency and orientation (Adjamian et al., 2008; Swettenham et al., 2009; Muthukumaraswamy and Singh, 2013). Thus, gamma oscillations in sensory areas appear to play a specific role in the encoding of stimulus features.

Gamma band activity has also been observed in motor and premotor areas from intracranial depth electrode recordings (Szurhaj et al., 2006) and surface electrocorticogram (ECoG) recordings (Pfurtscheller et al., 2003; Brovelli et al., 2005; Miller et al., 2007). Crone and colleagues (Crone et al., 1998) reported increased gamma activity in the ECoG in awake patients performing sustained muscle contractions. While movement-related gamma band synchronization has been reported in the scalp-recorded EEG (Ball et al., 2008; Darvas et al., 2010) such low-amplitude, high-frequency brain activity is highly susceptible to contamination by electromyographic (EMG) activity from scalp, jaw and neck muscles (Whitham et al., 2007) or even microsaccade-generated spike potentials (Yuval-Greenberg et al., 2008). MEG is much less susceptible to such volume conduction artifacts, and thus an ideal method for studying high-frequency cortical activity. However, caution should still be used in interpreting movement-locked gamma oscillations in both EEG and MEG data, particularly if such activity is similar in its frequency profile to simultaneously occurring EMG activity (Muthukumaraswamy, 2013). This can be particularly problematic for speech and orofacial movements, in which case it would be prudent to include EMG surface recordings of scalp muscle activity (Gross et al., 2013). Alternatively, source analysis methods such as minimum-variance beamforming can be used to optimally separate brain and muscle activity, although sources that localize to posterior or inferior frontal locations may still be affected by activity from nearby muscles and should be scrutinized for EMG contamination.

## Motor cortex gamma oscillations

There is abundant evidence that overt movements elicit a specific form of narrow-band, transient oscillatory activity in the range of 70–80 Hz in the region of the primary motor cortex. This “finely-tuned gamma” (Jenkinson et al., 2013) is distinguished from the spectrally broader, induced gamma band oscillations that are typically associated with perceptual or cognitive processes, and is strongly associated with the occurrence of overt movements. Most significantly, narrowly tuned gamma oscillations with similar timing and frequency are observed within various subcortical structures from depth electrodes implanted in patients for the treatment of various motor disorders, including the subthalamic nucleus (Amirnovin et al., 2004; Alegre et al., 2005; Androulidakis et al., 2007; Lalo et al., 2008) globus pallidus (Tsang et al., 2012a) and thalamus (Brucke et al., 2013). Using beamformer source analysis of MEG recordings, we were able to directly observe narrowly tuned gamma band oscillations in motor cortex in healthy subjects during movements of the upper and lower limbs (Cheyne et al., 2008). In comparison to lower frequency beta and mu band oscillations, which are observed bilaterally in MI, motor cortex gamma was highly lateralized to the contralateral primary motor cortex and somatotopically organized. These transient increases in high gamma with movement, sometimes referred to as gamma event-related synchronization (gamma ERS), have been replicated in a number of other MEG studies (Dalal et al., 2008; Tecchio et al., 2008; Muthukumaraswamy, 2010; Hinkley et al., 2011; Gaetz et al., 2013).

Muthukumaraswamy (2010) studied the influence of various kinematic and task parameters on motor cortex gamma, and found that both cued and voluntary movements elicited brief 200–300 ms bursts of gamma activity, even when followed by sustained isometric contractions. Similarly, repetitive movements generated multiple gamma bursts with each movement. Importantly, gamma oscillations did not appear to be simply due to movement-induced somatosensory feedback, since individual gamma bursts were observed for active, but not passive movements at the same rate. Gamma activity preceding the onset of movement has been observed for complex tasks (Gaetz et al., 2013) and self-paced as opposed to visually cued movements in subcortical recordings (Tsang et al., 2012a) and we have also noted variable timing of gamma bursts when inspecting single trial data. Sustained gamma activity has been reported for continuous tracking movements (Kennedy et al., 2011), although we have observed a marked decrement in motor cortex gamma for fast (>1 Hz) movement rates, indicating that it may be reduced during rhythmic movement (unpublished observations). Bilateral gamma oscillations are elicited by lip movements (Bells et al., 2007) and gamma oscillations have been observed in frontal eye-fields during saccadic eye-movements (Hinkley et al., 2011). Movement-related gamma activity has also been shown to be present in children and adolescents (Gaetz et al., 2010; Wilson et al., 2010; Huo et al., 2011), including children as young as 3.5 years of age (Cheyne, 2013).

Thus, high-frequency, narrowly tuned gamma oscillations are elicited by many types of movement, are strongly linked to movement onset, and appear to reflect activation of neural populations in primary motor cortex. Somatosensory stimulation can also induce high-frequency activity in somatosensory areas (Bauer et al., 2006) raising the possibility that there may be some contributions from SI due to movement-induced sensory feedback. However, movement-related gamma has been consistently localized to regions of the precentral gyrus using beamformer analysis of MEG data, which has been shown to discriminate precentral and postcentral sources of movement-related activity (Cheyne et al., 2006; Jurkiewicz et al., 2006; Cheyne, 2013). This, combined with the fact movement-related gamma can be observed prior to movement onset, and is not elicited by passive movements alone, suggests that it plays an important role in the generation or monitoring of motor output.

## Motor gamma and individual differences

**Figure 1:**
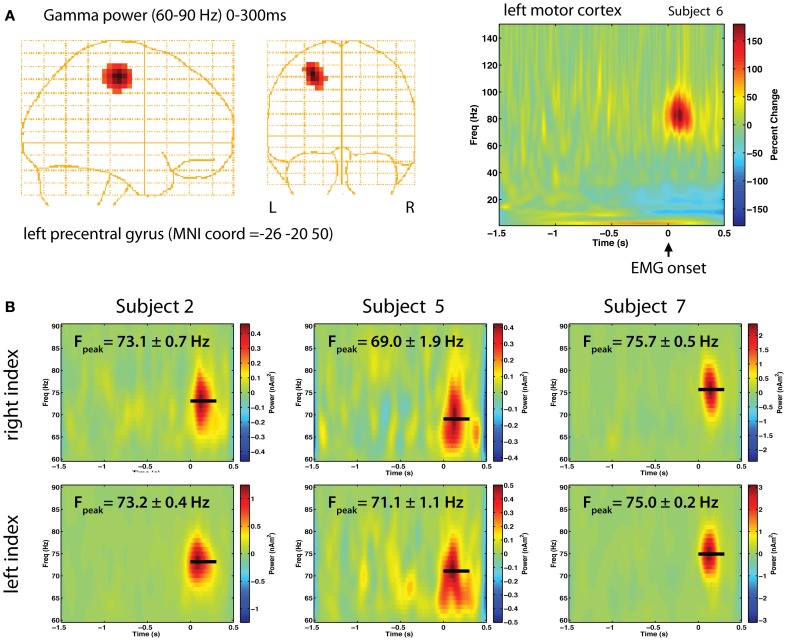
**(A)** Gamma band responses during right finger abductions in a right-handed subject. Synthetic Aperture Magnetometry (SAM) source localization was used to localize the generator of gamma power in right motor cortex, transformed to MNI coordinates with 4 mm resolution (left). A time-frequency plot of the source activity (right) over the range of 1–150 Hz shows a brief burst of gamma band activity around 80 Hz immediately following EMG onset (*t* = 0 s) as a percent change above baseline (−1.5 to −1 s). **(B)** Time-frequency analysis of source activity from contralateral motor cortex for left and right index finger movements in three selected right-handed subjects, showing the peak frequency (*F*_peak_) and standard error in each subject, estimated from 5000 bootstrap resampling (with replacement) of the single trial time-frequency estimates. Beamformer source analysis was carried out using the BrainWave analysis toolbox (http://cheynelab.utoronto.ca).

**Figure 2:**
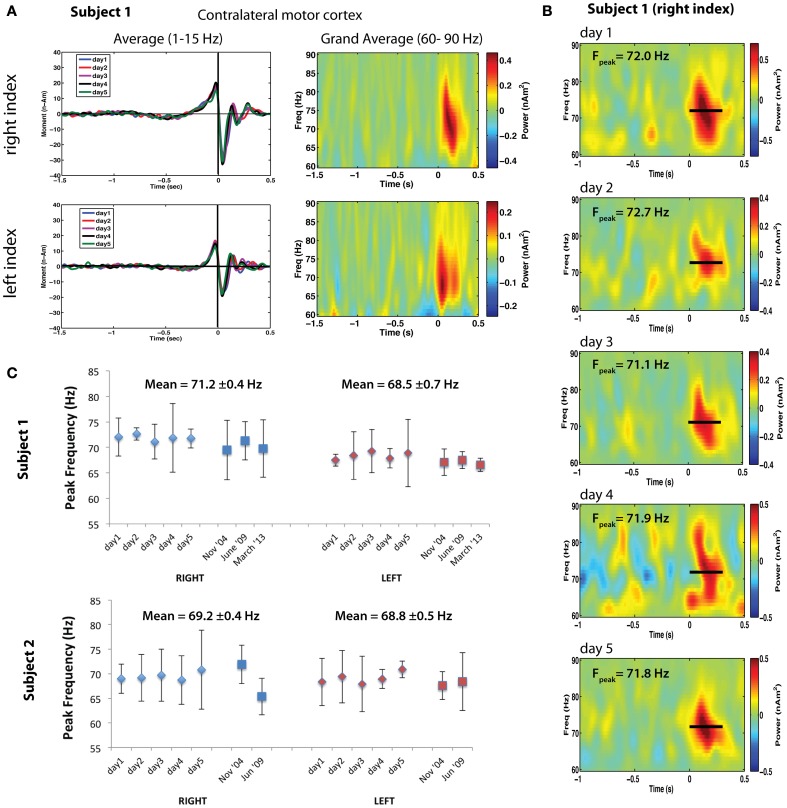
**(A)** Left: Averaged motor fields for all 5 recording sessions for left and right index finger movements (time-locked to button press). Right: Corresponding time-frequency analysis of the single trial power from 60 to 90 Hz for the peak locations of gamma activity. **(B)** Bootstrapped mean time-frequency plots for the 5 repeated sessions for right index finger movements. *F*_peak_ indicates the mean peak frequency ± standard error. **(C)** Peak motor cortex gamma frequencies for right index finger (RIGHT, blue markers) and left index finger (LEFT, red markers) movements repeated for 5 sessions over a one-week period for two subjects. Sessions taken at different time intervals separated by approximately 4–5 years are also shown. Each data point indicates the bootstrap resampled mean peak frequency, using 5000 re-samplings of two-thirds of the single trial time-frequency estimates. Error bars indicate the 95% confidence intervals based on the bootstrap standard error.

In our earlier study, we noted a marked similarity in the strength, frequency and duration of gamma oscillations observed in the left and right motor cortex for movements of the same body part in individual subjects (Cheyne et al., 2008). A re-analysis of these data is presented in Figure 1. Using the synthetic aperture magnetometry (SAM) beamformer (Robinson and Vrba, 1999) we generated 4 mm images of changes in gamma power (60–90 Hz) relative to pre-movement baseline. Figure 1A shows a gamma increase localized to a focal location in the contralateral primary motor cortex in one subject, and the corresponding Morlet-wavelet based time-frequency reconstruction of source activity (width = 20 cycles), demonstrating an extremely narrow-band power increase centered around 80 Hz. We performed bootstrap resampling to estimate the standard errors (SEs) of peak gamma frequency across trials (Muthukumaraswamy and Singh, 2013) and found that peak frequency was highly similar between hemispheres, varying by less than 1 Hz within individual subjects (Figure 1B). As indicated by the relatively small SEs, gamma frequency was stable across trials, with each subject showing characteristic peak gamma frequency. As reported in the initial study, a high degree of left-right symmetry was also observed for bicep contractions and foot dorsiflexions, with lower gamma frequency for lower compared to upper limb movements. As shown in Figure 2B, the absolute strengths of gamma bursts are small, ranging from 0.5 to 2.0 nAm^2^. Using data simulations we confirmed that the latter would correspond to an oscillating dipole source of about 5 nAm in source strength, generating peak-peak amplitudes at the sensors on the order of about 50 femtoTesla, making such signals difficult to observe in the raw data.

The foregoing observations suggest that motor cortex gamma oscillations are finely tuned in frequency, show similarities for different movement types within individuals, and frequency differences across individuals. This implies that each individual has a characteristic pattern of motor gamma activation—a gamma band “fingerprint”—for specific types of motor output. If so, these patterns should be replicable across recording sessions. To test this hypothesis, we retrospectively analyzed data from two right-handed adults (including one of the authors) in which MEG recordings during left and right self-paced index finger movements (button presses) had been repeated in separate sessions at the same time each day, over a five-day period. In addition, these measurements were available in both subjects recorded ~5 years earlier, and in one subject 4.5 years later. Figure 2 presents an analysis of motor cortex gamma activity across different sessions for both subjects. Averaged time courses of source activity in contralateral MI, computed using an event-related beamformer (Cheyne et al., 2006), confirmed that the subject's performance was highly consistent across sessions (Figure 2A). Time-frequency plots of source power in MI for left and right movements show gamma bursts beginning at movement onset and lasting ~300 ms (Figure 2A right). Mean peak frequency and SEs for each hemisphere and session were estimated using bootstrap resampling of single trial time-frequency estimates and compared over all sessions (a total of 8 sessions for Subject 1 and 7 sessions for Subject 2). Figure 2B shows time-frequency plots for the right hand condition in Subject 1, with peak gamma frequency often varying by less than 1 Hz between sessions. Figure 2C shows the peak frequency over all sessions for both subjects, demonstrating stability over multiple sessions. Peak gamma frequency was lower for left compared to right index finger movements across sessions for Subject 1, with mean peak frequency (±SE) of 71.24 ± 0.38 Hz for right finger movements and 68.47 ± 0.71 Hz for left finger movements (*p* < 0.005, paired *t*-test). Interestingly, the latency of gamma peak frequency was also earlier for left finger movements (79.26 ± 9.4 ms) compared to right finger movements (151.1 ± 7.5 ms) across all sessions (*p* < 0.0001, paired *t*-test). For Subject 2, peak frequency was also consistent for both left and right index finger movements across repeated sessions, with almost identical mean gamma frequency for left (68.2 ± 0.4 Hz) and right (69.8 ± 0.5 Hz) finger movements (*p* = 0.39, paired *t*-test). Gamma latency was also not significantly different for left and right movements in this subject. Although preliminary, these results suggest that individuals demonstrate highly replicable peak gamma frequencies in motor cortex for the same movements, and that these are stable over long periods of time, indicative of an inter-individual characteristic frequency of motor cortex gamma oscillations.

## The functional role of motor cortex gamma oscillations

The role of high gamma oscillations in motor control is unknown. It is clear that these oscillations are markedly similar in frequency and latency to those observed in the basal ganglia and other subcortical motor structures, which have reciprocal connections with motor cortex (Shepherd, 2013). Coherence between oscillations in MI and STN are well established for the lower beta band (Brown, 2003; Lalo et al., 2008). However, such long-range synchronization for gamma band activity is incompatible with conventional views that higher frequency gamma band oscillations are likely generated by short-range neuronal circuits within the cortex, most likely involving inhibitory interneurons (Kopell et al., 2000; Whittington and Traub, 2003). The role of local inhibitory feedback in cortical gamma generation is partly supported by the observation that both VI and MI gamma frequency has been shown to be correlated with *in-vivo* measures of the inhibitory neurotransmitter GABA (Muthukumaraswamy et al., 2009; Gaetz et al., 2011) although a more recent MEG study found that only beta, and not gamma oscillations in MI were modulated by a GABA_A_ receptor agonist (Muthukumaraswamy et al., 2012). In addition, recent evidence suggests that GABAergic mechanisms may also reflect long-range connectivity within the brain (Caputi et al., 2013). Litvak et al. (2012) applied coherence and Granger causality methods to movement-related gamma oscillations measured simultaneously in MI and STN in Parkinson patients to estimate long-range coupling and its directionality between these structures. They found evidence that the STN is driven by MI in the beta band and a weak effect for gamma activity showing the opposite directionality (STN phase-leading cortex). It is unclear what mechanisms might be involved in the latter, since STN activity would have to propagate via globus pallidus and thalamus back to MI, although there is recent evidence of direct projections from STN to MI (Degos et al., 2008). Thus, the role long-range cortical-striatal loops in the neurogenesis of co-occurring gamma bursts in MI and subcortical structures remains to be fully resolved.

The results of our reproducibility experiment, although requiring further replication, provide evidence that gamma band frequency is highly consistent within subjects (and hemispheres) over time, suggesting that an individual's motor cortex gamma is tuned to a preferred frequency, at least for the same movement. Interestingly, a recent MEG study demonstrated that the frequency of visual cortex gamma was positively correlated with the surface area of visual cortex stimulated, suggesting that inter-individual variations in cortical anatomy determine gamma oscillation frequency. A similar mechanism might underlie motor cortex gamma given its dependence on the type of movement performed. For example, upper limb movements should activate a larger region of MI than lower limb movements, the latter showing significantly lower gamma frequency (Cheyne et al., 2008). However, this seems a less plausible explanation for the marked left-right symmetry of motor cortex gamma frequency, which, somewhat speculatively, may be more likely attributed to symmetrical conduction times or distances between cortical and subcortical structures, if not due to synchronization by common subcortical pathways. Our results do not lend support to previous observations of a decrease in motor gamma frequency with age (Muthukumaraswamy et al., 2009; Gaetz et al., 2011) as we observed no consistent changes in peak gamma frequency over a several year period in either subject. More detailed longitudinal studies may be required to establish the role of individual differences such as age in the tuning of motor cortex gamma frequencies.

These results also suggest that the facilitative effect of gamma band stimulation on movement might be enhanced by the use of individual's preferred gamma frequency. Tsang and colleagues (Tsang et al., 2012b) found that individualized gamma stimulation frequencies improved symptoms in Parkinson patients equally well as standard HF stimulation, although they did not directly compare non-preferred gamma frequencies. Similarly, transcranial alternating current stimulation of the motor cortex at exactly 70 Hz was shown to have a facilitative effect on movement (Joundi et al., 2012) and such effects might be enhanced by stimulation at the individual's preferred gamma frequency, although such effects will likely be effector specific.

## Conclusions

What is the functional meaning of high-frequency gamma oscillations in normal motor control? The presence of gamma oscillations during virtually all types of movement supports the general finding they have a functional, or even facilitative role in motor output. We initially speculated that gamma activity at movement onset reflected a “disinhibition” of cortico-basal ganglia motor circuits via projections from MI to STN necessary to initiate movement (Cheyne et al., 2008). However, evidence that gamma activity in MI phase-lags coherent activity in subcortical structures (Lalo et al., 2008; Litvak et al., 2012) has led to speculations that motor cortex gamma might be the result of propagation of activity through the thalamus, which is also driven by brainstem nuclei related to arousal (Brucke et al., 2012). This, combined with the observation that subcortical movement-evoked gamma scaled with movement amplitude, led these authors to propose that motor cortex gamma synchronization reflects a non-specific momentary state of arousal during (or enabling) the initiation of movement, reflecting response “vigour” (Jenkinson et al., 2013). However, a recent study showed that machine learning algorithms were able to distinguish different patterns of activity recorded from ECoG in patients sufficiently to control a prosthetic arm, providing evidence that signatures for different movements may exist in the motor gamma response (Yanagisawa et al., 2012).

An alternative hypothesis, not entirely incompatible with the above observations, is that motor gamma oscillations may reflect efference copy mechanisms during movement that underlie our attention to movement execution. A recent theory of basal ganglia function proposed that two distinct cortico-striatal loops provide a mechanism for the computation of error signals between planned and executed movements (Morita et al., 2012). In this model, preparatory signals are transferred from direct pathway circuits to one involving cortical pyramidal cells that “drive the indirect pathway with signals representing the action currently being executed” (Shepherd, 2013, p. 284). In this context, cortical gamma oscillations may represent signals relayed upstream from the basal ganglia regarding planned movements, and constitute part of a network involved in the comparison of motor intentions with sensory feedback, allowing for discrimination between motor “plans” and actual executed movements. Thus, motor cortex gamma may not be imperative for actual motor control, but may provide a temporal encoding of planned movements, contributing to our conscious awareness of having performed an intended movement. This would also be compatible with the observation that motor gamma seems to encode specific features of executed movements, yet is variable in amplitude and timing for individual movements, and the degree to which it is correlated with movement performance. This, in turn, may reflect fluctuations in our awareness of our actions on a moment-to-moment basis. Further studies of the relationship of cortical gamma oscillations to motor attention and performance may help further unravel the neurogenesis of cortical gamma oscillations and their role in motor control.

### Conflict of interest statement

The authors declare that the research was conducted in the absence of any commercial or financial relationships that could be construed as a potential conflict of interest.
